# Prohibitin modulates periodontium differentiation in mice development

**DOI:** 10.3389/fcell.2024.1369634

**Published:** 2024-05-02

**Authors:** Yam Prasad Aryal, Song-Yi Han, Bandana Rana, Sanjiv Neupane, Tae-Young Kim, Elina Pokharel, Jung-Hong Ha, Jae-Kwang Jung, Chang-Hyeon An, Ji-Youn Kim, Hitoshi Yamamoto, Youngkyun Lee, Seo-Young An, Jo-Young Suh, Jae-Young Kim, Wern-Joo Sohn

**Affiliations:** ^1^ Department of Biochemistry, School of Dentistry, IHBR, Kyungpook National University, Daegu, Republic of Korea; ^2^ Department of Biological Sciences and Biotechnology, College of Natural Sciences, Chungbuk National University, Cheongju, Republic of Korea; ^3^ Department of Periodontology, School of Dentistry, IHBR, Kyungpook National University, Daegu, Republic of Korea; ^4^ Department of Biochemistry and Cell Biology, Stony Brook University, Stony Brook, United Sates; ^5^ Department of Conservative Dentistry, School of Dentistry, IHBR, Kyungpook National University, Daegu, Republic of Korea; ^6^ Department of Oral Medicine, School of Dentistry, IHBR, Kyungpook National University, Daegu, Republic of Korea; ^7^ Department of Oral and Maxillofacial Radiology, School of Dentistry, IHBR, Kyungpook National University, Daegu, Republic of Korea; ^8^ Department of Dental Hygiene, Gachon University, Incheon, Republic of Korea; ^9^ Department of Histology and Developmental Biology, Tokyo Dental College, Toky, Japan; ^10^ College of K-Biohealth, Daegu Haany University, Gyeongsan, Republic of Korea

**Keywords:** periodontium, differentiation, functional evaluation, alveolar bone process, microinjecfion

## Abstract

**Introduction:** Prohibitin (PHB) is an essential scaffold protein that modulates signaling pathways controlling cell survival, metabolism, inflammation, and bone formation. However, its specific role in periodontium development remains less understood. This study aims to elucidate the expression pattern and function of PHB in periodontium development and its involvement in alveolar bone formation.

**Methods:** Immunolocalization of PHB in the periodontium of postnatal (PN) mice were examined. *Phb* morpholino was micro-injected into the right-side mandible at PN5, corresponding to the position where the alveolar bone process forms in relation to the lower first molar. The micro-injection with a scramble control (PF-127) and the left-side mandibles were used as control groups. Five days post-micro-injection, immunohistochemical analysis and micro-CT evaluation were conducted to assess bone mass and morphological changes. Additionally, expression patterns of signaling molecules were examined following *Phb* downregulation using 24-h *in vitro* cultivation of developing dental mesenchyme at E14.5.

**Results:** The immunostaining of PHB showed its localization in the periodontium at PN5, PN8, and PN10. The *in vitro* cultivation of dental mesenchyme resulted in alterations in Bmps, Runx2, and Wnt signalings after *Phb* knock-down. At 5 days post-micro-injection, *Phb* knocking down showed weak immunolocalizations of runt-related transcription factor (RUNX2) and osteocalcin (OCN). However, knocking down *Phb* led to histological alterations characterized by decreased bone mass and stronger localizations of Ki67 and PERIOSTIN in the periodontium compared 1 to control groups. The micro-CT evaluation showed decreased bone volume and increased PDL space in the *Phb* knock-down specimens, suggesting its regulatory role in bone formation.

**Discussion:** The region-specific localization of PHB in the margin where alveolar bone forms suggests its involvement in alveolar bone formation and the differentiation of the periodontal ligament. Overall, our findings suggest that *Phb* plays a modulatory role in alveolar bone formation by harmoniously regulating bone-forming-related signaling molecules during periodontium development.

## Introduction

The periodontium develops from mesenchymal precursor cells within the dental follicle, consisting of fibroblasts, osteoblasts, and cementoblasts, which differentiate into the periodontal ligament fibers, alveolar bone, and cementum respectively (Grobstein, 1955). Numerous studies have explored the developmental processes of the periodontium, aiming to examine the precise signaling pathways involved in these mechanisms (Souza et al., 2012; Zhang et al., 2017; Wei et al., 2021). The fine-tuning of harmonized signaling regulations in periodontium development is considered essential for achieving both structural and functional regeneration of the periodontium as a functional unit.

To date, primary cell lines, *in vitro* organ cultivation, and genetically manipulated mice have served as the primary experimental systems for evaluating signaling regulations in periodontium differentiation (Luan et al., 2006; Fujii et al., 2008; Neupane et al., 2020). However, these systems are inadequate to fully represent and understand the complexities of the *in vivo* condition, given that the periodontium comprises multiple cell types and exhibits intricate interactions during its development (Stepaniuk and Hinrichs, 2013). Notably, the lack of suitable *in vitro* and experimental animal model systems for evaluating detailed signaling regulations underlying periodontium differentiation remains a challenge. Moreover, the study of the alveolar bone process, a dynamic and functional structure supporting the teeth, has been insufficient. These incomplete understandings of periodontium differentiation might impede the development of techniques for functionally regenerating the periodontium, encompassing the alveolar bone, periodontal ligament, and cementum.

Prohibitin (PHB) constitutes a highly conserved and widely expressed family of proteins, known as the prohibitin domain family, implicated in various processes such as transcriptional regulation, cell proliferation, development, and mitochondrial function (Mishra et al., 2005; Supale et al., 2013; Della-Flora Nunes et al., 2021). Extensive studies have elucidated the therapeutic roles of PHB as a cell proliferation inhibitor in various cancers (Theiss and Sitaraman, 2011), and its involvement in rodent uterine development and ovarian cell differentiation has been suggested (Thompson et al., 1999; Thompson et al., 2001; He et al., 2011). Additionally, PHB has been identified as a strong binding protein for anti-resorptive compounds, inhibiting osteoclast differentiation (Lee et al., 2015). Particularly, lower expression of PHB has been associated with the regulation of osteogenesis-related signaling molecules, leading to increased proliferation and formation of osteoblasts (Zhu et al., 2010). Based on these prior reports, we hypothesize that PHB plays a crucial role in the intricate processes involved in the structural and functional formation of the periodontium, especially in the formation of the alveolar bone process and periodontal ligament. In this study, we employed a previously established micro-injection model (An et al., 2017) and an *in vitro* tissue culture model system to analyze the precise developmental mechanisms mediated by the function of PHB during periodontium differentiation.

## Materials and methods

### Animals

All experiments involving animals were performed in accordance with the guidelines of the Kyungpook National University, School of Dentistry, Intramural Animal Use and Care Committee (KNU-2020-0107). Adult ICR mice were housed in optimum conditions, including room temperature (22°C ± 2°C), 55 % ± 5% humidity and artificial illumination with lights on from 05:00 to 17:00 h, with access to food and water *ad libitum*. Postnatal mice at day 5 (PN5) were used for micro-injection experiment. For *in vitro* tissue cultivations, 5 pregnant mice were sacrificed at embryonic day 14.5 (E14.5) and at least 50 embryos were used.

### Micro-injection of signaling molecules

*Phb* morpholino (1 µM with 15% PF127; Gene Tools, LLC, United States) and carrier control (15% PF127) were micro-injected on right side of the PN5 mandible as described previously (Supplementary Figure S1) (An et al., 2017). Our result showed successful microinjection with knockdown of *Phb* in the periodontium region (Supplementary Figures S1, S2). The left side of mandibles were used as control. At least 12 newborn ICR mice (PN5) were examined for each group (control and experimental). The concentrations were decided with the previous report (Chang et al., 2011). Over 90% of the postnatal mice survived after microinjection and to ensure their normal behavior, we monitored the cages every 24 h. After 5 days from micro-injections, mice were sacrificed, and mandibles were harvested for morphological and immunohistochemical analysis.

### Specimen preparation for micro computed tomography

The harvested mandible specimens were fixed with 4% formaldehyde at 4°C and then subjected to micro-computed tomography (micro-CT) analysis (SkyScan1272; 166μA, 60kV; Bruker, United States). Computed tomography (CT) was used to study the 3D structure of hard tissue in periodontium and allowed the selection of virtual parallel slices spaced by 7 μm planes. The image data from the scanned planes were subsequently reconstructed using N Recon software (SkyScan, United States).

### Histology and immunohistochemistry

Histological staining, including hematoxylin and eosin (H&E) and Masson’s trichrome (MTC), as well as immunostainings, were conducted following previously established protocols (An et al., 2017). Briefly, after deparaffinization and rehydration, the sections were processed for either H&E and MTC staining or processed with antigen retrieval for immunostainings. Non-specific antibodies were blocked using 1X western blocking solution (Germany, Mannheim, Roche; Ref. 11921673001). The primary antibodies directed against PROHIBITIN (Abcam, Cat. No. ab28172), PERIOSTIN (Abcam, Cat. No. ab14041), Ki67 (Neo Markers, Cat. No. RM-9106), RUNX2 (Abcam, Cat. No. ab192256) and Osteocalcin (Abcam, Cat. No. ab93876) were used. The secondary antibodies used in this study were biotinylated goat anti-rabbit IgG (Invitrogen, Waltham, MA, United States). The binding of the primary antibody to the sections were visualized by using a diaminobenzidinetetrahydrochloride (DAB) reagent kit (Zymed, Cat. No. 00-2014). All experiments were performed a minimum of three times.

### *In vitro* tissue cultivation and qPCR

The molar tooth germs at E14.5 were dissected and incubated in Dispase II (Roche, Germany) at 1.2 Unit/ml in PBS for 20 min. The tooth germs were rinsed in DMEM with 20% FBS for 10 min. For drop cultivation, mesenchymal tissue was prepared after removing epithelium and cultivated in DMEM containing 10% FBS and 1% Penicillin Streptomycin. For experimental group, 1 μM *Phb* morpholino and for control group, 0.01% DMSO were added into the medium for 24 h. The sequences of *Phb*-oligodeoxynucleotides (ODNs) were as follows: antisense AS-ODN 5′-AGA​TAC​GAG​GAA​GCT​GGC​TG-3′ and sense (S) ODN 5′-CAG​CCA​GCT​TCC​TCG​TAT​CT-3′. Total RNA extraction and cDNA synthesis for qPCR analysis were carried out using RNeasy^®^ Micro Kit (Germany, Qiagen; Cat. No. 74004) and Omniscript^®^ RT Kit (Germany, Qiagen; Cat No. 205111) respectively as described previously (Adhikari et al., 2021). The primers used in this study are listed in Supplementary Table S1. The data have been expressed as mean ± S.D. The mean expression levels of the experimental and control groups were compared using the Student’s t-test; *p < 0.05* was considered significant.

### Tartrate-resistant acid phosphatase staining

The osteoclast number was evaluated by staining the slide for TRAP (Sigma, MO, United States), as described previously (Adhikari et al., 2021). TRAP-positive cells with three or more nuclei were counted as multinuclear osteoclasts.

### Statistical analysis

ImageJ software (http://imagej.net/) was used to count the immunostaining positive cells as described in previous report (Neupane et al., 2020). The number of PHB, Ki67, RUNX2, and Osteocalcin positive cells in the DAB-stained sections were counted in the defined area of periodontium. Data were represented as ± standard deviations and the mean was determined by comparing control and experimental groups using Student’s t-test. *p < 0.05* indicates significance. On the other hand, the intensity of immunostaining against Periostin were quantified as -:none, +: exist, ++: strong, +++: strongest because of their broad nature of localization patterns outside the nucleus.

## Results

### Localization of PHB in the developing periodontium

**FIGURE 1:**
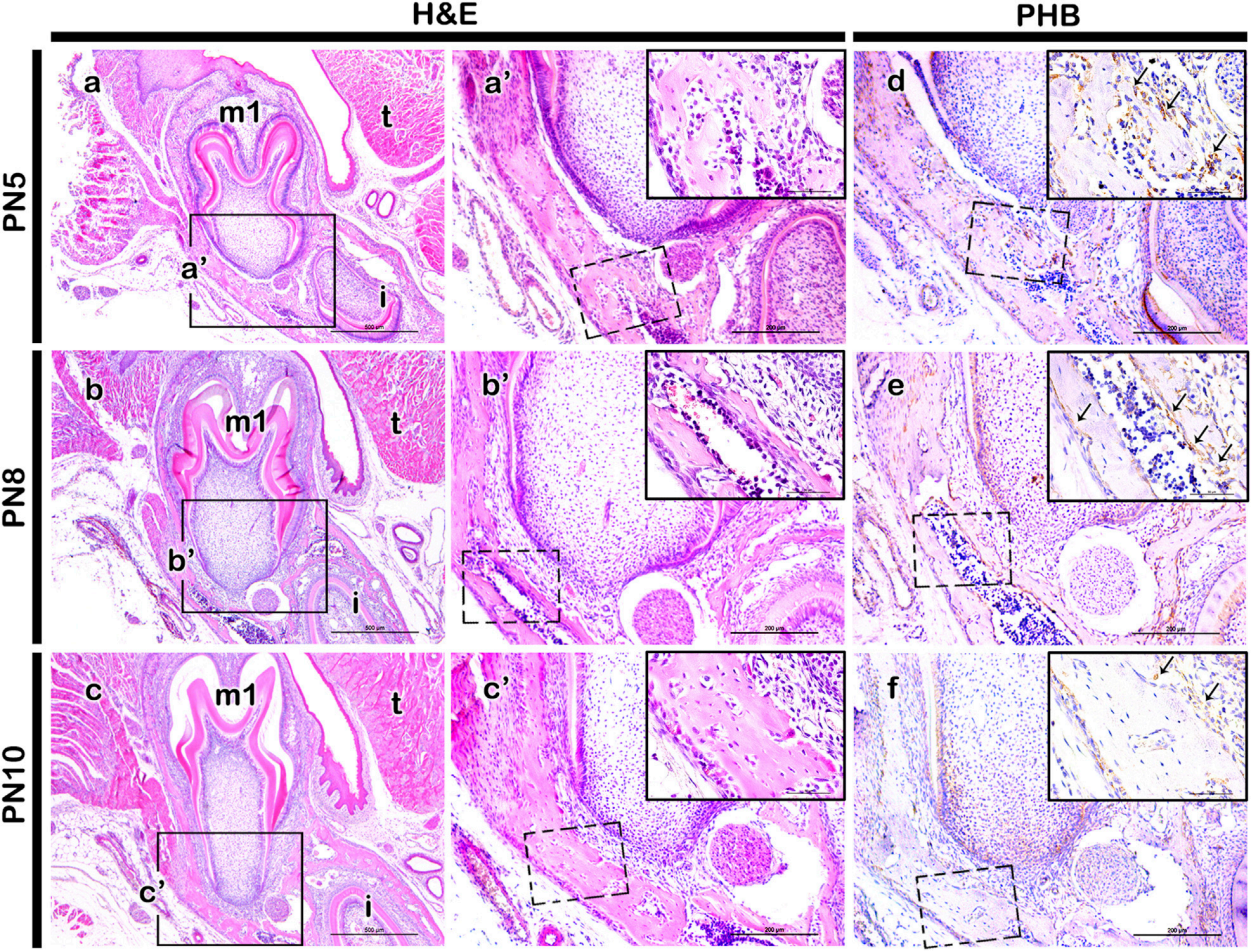
Localization pattern of PHB in the developing periodontium. H&E staining showing developing periodontium and adjacent tissue at PN5, PN8 and PN10 mandible **(A–C)**. In the mesial root forming region, PHB localization is observed in the periodontium at PN5, PN8, and PN10 **(D–F)**. At PN5, PHB is broadly localized in the developing periodontium **(D)**. By PN8, a stronger positive reaction of PHB is observed in the alveolar bone forming regions **(E)**. However, a weaker localization pattern of PHB is detected at PN10 **(F)**. Square boxes in a-c indicate enlarged view in **(a’-c’)**, respectively. Dotted rectangles indicate the magnified area presented as inset images in the top right of the respective figure **(a’-c’)**, **(D–F)**. H&E; hematoxylin and eosin, PHB; prohibitin, m1; molar 1, i; incisor, t; tongue. *Scale bars*: 500 µm **(A–C)**, 200 µm **(a’-c’)**, **(D–F)**, 50 µm (images in the inset).

Frontal sections from postnatal mice (PN5, PN8, and PN10) were examined to analyze the tissue forming the alveolar bone process within the mesial root forming region (Figures 1A–C). At PN5, the initiation of tooth root elongation was observed with the growth of Hertwig’s epithelial root sheath (HERS) (Figure 1A-a’). At PN8, the elongating root showed increased length accompanied by a rise in fibroblast cell count and thickening of the mandibular bone (Figure 1B-b’). At PN10, there was a notable increase in the thickness of the fibroblast cell layer between the tooth and the mandibular bone. This stage allowed for easier identification of periodontal ligament-like and alveolar bone process forming tissues (Figure 1C-c’). Consequently, the elongation of the tooth root made the alveolar crest and tooth crown more distinguishable (Figure 1C’). Immunolocalization of PHB revealed its presence in the periodontium forming tissues at PN5 (Figure 1D), with a stronger localization observed at PN8 (Figure 1E). By PN10, the localization was comparatively weaker than at PN5 and PN8 (Figure 1F).

### Evaluation of gene expression patterns using a drop cultivation method

**FIGURE 2:**
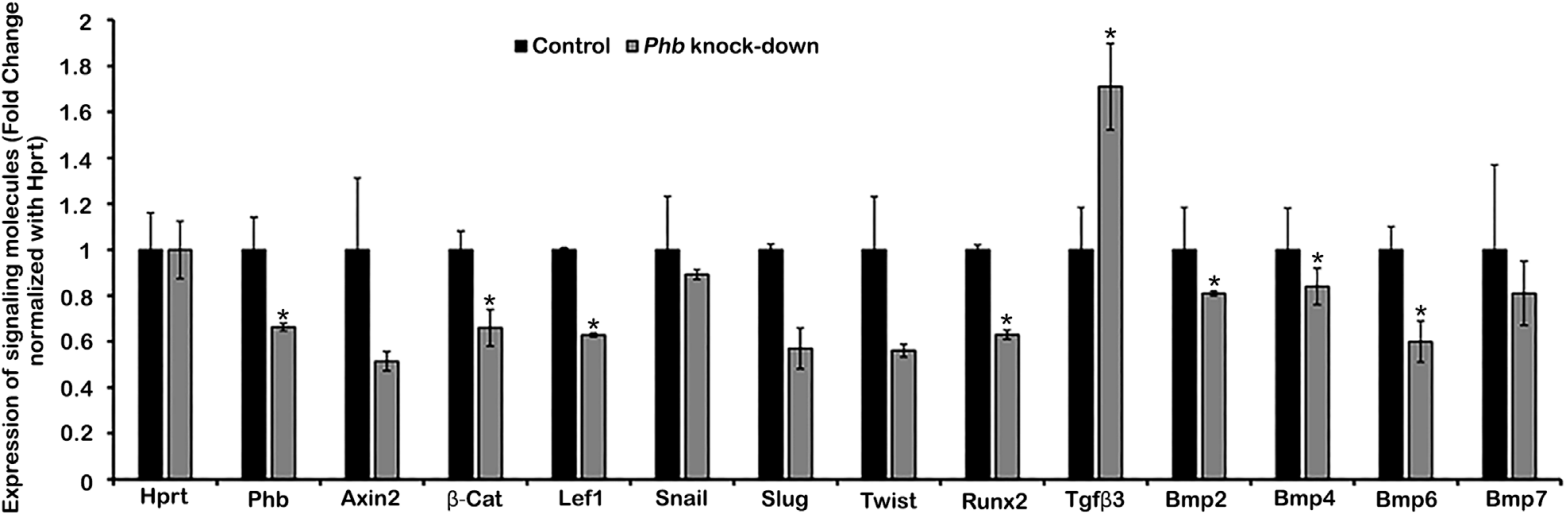
Evaluation of differential expression patterns of candidate signaling molecules. qPCR showing altered expression patterns of signaling molecules after knocking down *Phb*. Expression levels are normalized by Hprt. * denotes *p < 0.05*.

To elucidate the signaling regulation modulated by *Phb*, we utilized the drop cultivation method, as previously described (An et al., 2017). Developing dental meenchymal cells at E14.5 were harvested and cultivated for 24 h with or without *Phb* knock-down (Figure 2). RT-qPCR was employed to examine the altered expression patterns of known signaling molecules expressed in periodontium development (Figure 2). Specifically, we investigated the expression patterns of Wnt- and TGFβ/BMP-related signaling molecules, including *Axin-2, β-Catenin, Lef1, Slug, Twist, Tgfβ2, Runx2, Bmp2, Bmp4, Bmp6,* and *Bmp7* (Figure 2). These molecules are well-known for their involvement in mesenchymal tissue differentiation during organogenesis (Kim et al., 2007; Beederman et al., 2013). The knock-down of *Phb* during the *in vitro* cultivation of dental mesenchymal cells resulted in altered expressions of these signaling molecules. Particularly, the expression patterns of *β-Catenin, Lef1, Runx2, Bmp2, Bmp4* and *Bmp6* were significantly downregulated after *Phb* knock-down (Figure 2).

### Morphological alterations in periodontium after knocking down *Phb*

**FIGURE 3:**
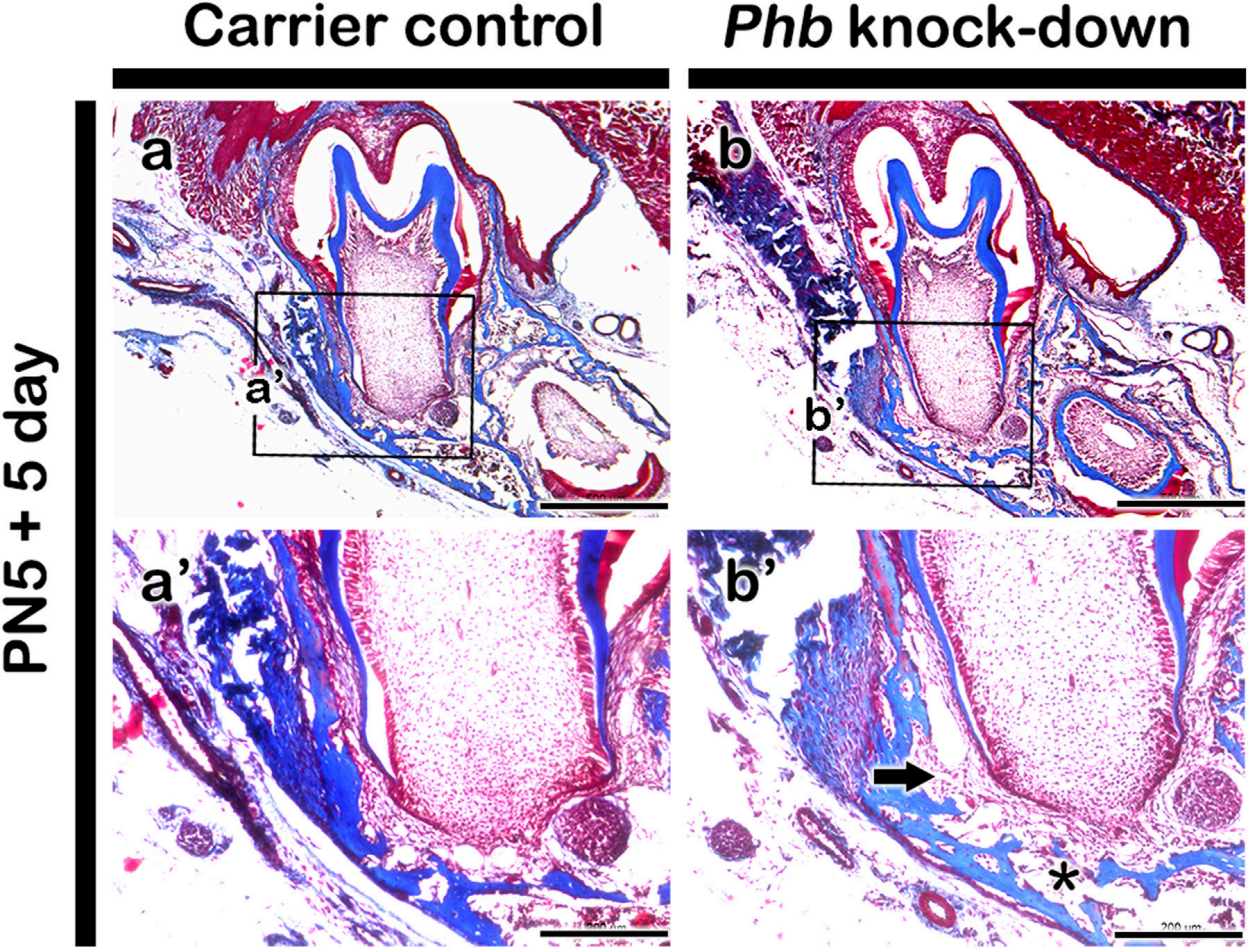
Histological analysis of periodontium after micro-injection of Phb morpholino. Compared with carrier control **(aa’)**, MTC staining shows increased PDL space (arrow) and decreased alveolar bone (*) after *Phb* knocking down **(bb’)**. Rectangular boxes **(A,B)** indicate the magnified regions presented in **(a’-b’)**. *Scale bars*: 500 μm **(A,B)**, 200 μm **(a’-b’)**.

To understand the morphological alterations, we examined the histomorphology of the carrier control and experimental groups in the periodontium of the first lower molar tooth following *Phb* morpholino treatment for 5 days (Figures 3aa′-bb′). MTC staining unveiled developing structures of alveolar bone, periodontal ligament, and cementum-forming tissues in the buccal side of the lower molar tooth root-forming region in both carrier control and *Phb* micro-injected specimens (Figures 3aa′-bb′). Nevertheless, when juxtaposed with the control group, *Phb* knock-down specimens exhibited an increased PDL (periodontal ligament) space, accompanied by an evident reduction in alveolar bone thickness (Figure 3B-b’). Specifically, these morphological changes in the developing periodontium were primarily noticeable in the alveolar bone-forming region rather than the cementum-forming region (Figure 3bb′).

### Alterations of cellular physiology in developing periodontium

**FIGURE 4:**
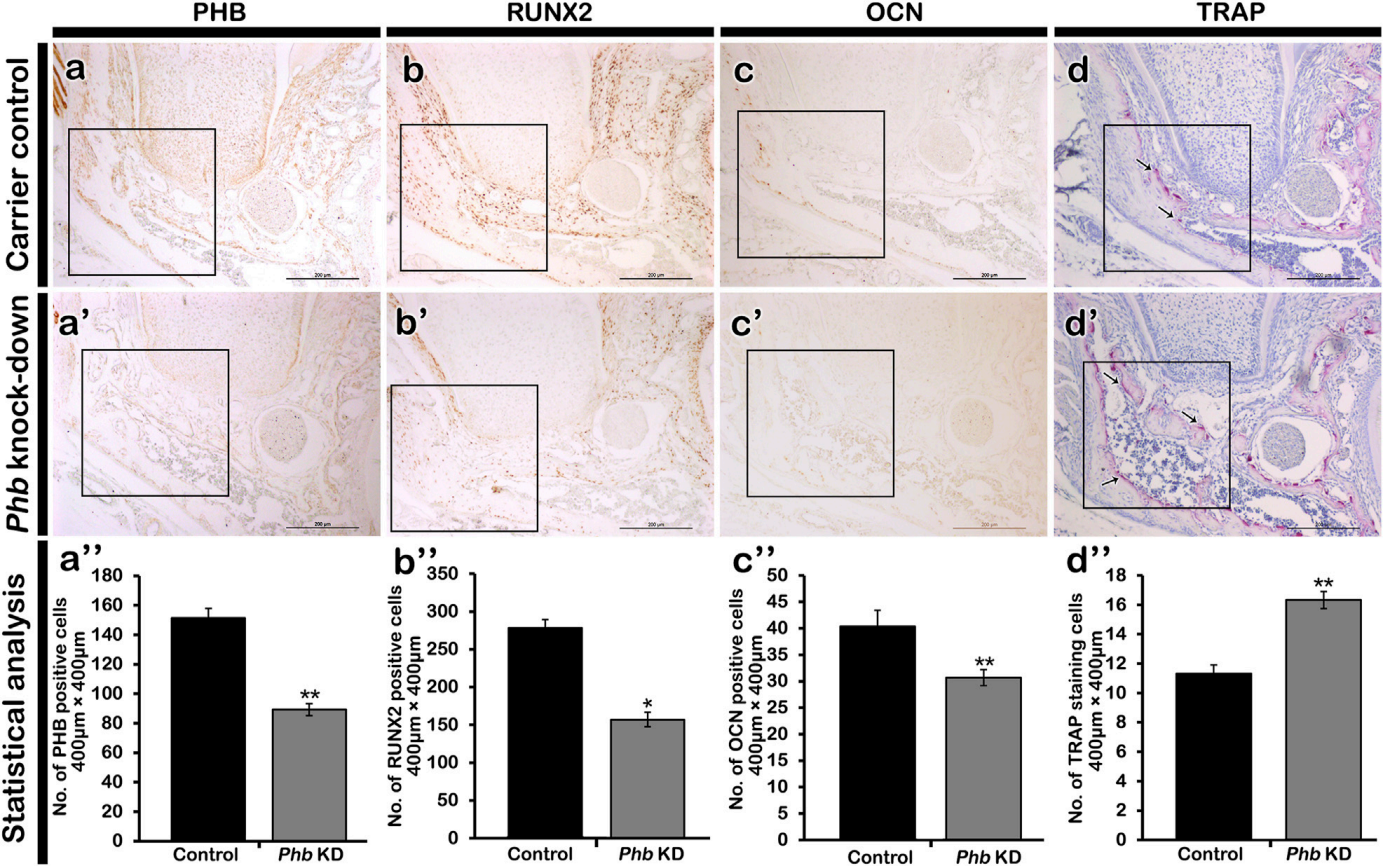
Altered localizations of bone forming factors. Immunolocalizations of PHB **(A)**, RUNX2 **(B)** and OCN **(C)**. Compared to control, the localizations of PHB, RUNX2 and OCN in the PDL forming region are decreased after knocking down of *Phb* **(aa’a”-cc’c”)**. However, the number of bone resorbing cells, osteoclasts are increased in the *Phb* knock-down specimen when compared to control **(d-d”)**, arrows. Quantification of immunopositive cells using ImageJ **(a”-d”)**. RUNX2; runt-related transcription factor, OCN; osteocalcin. * and ** indicate *p < 0.05* and *0.01,* respectively. *Scale bars*: 200 μm **(aa’-dd’)**.

**FIGURE 5:**
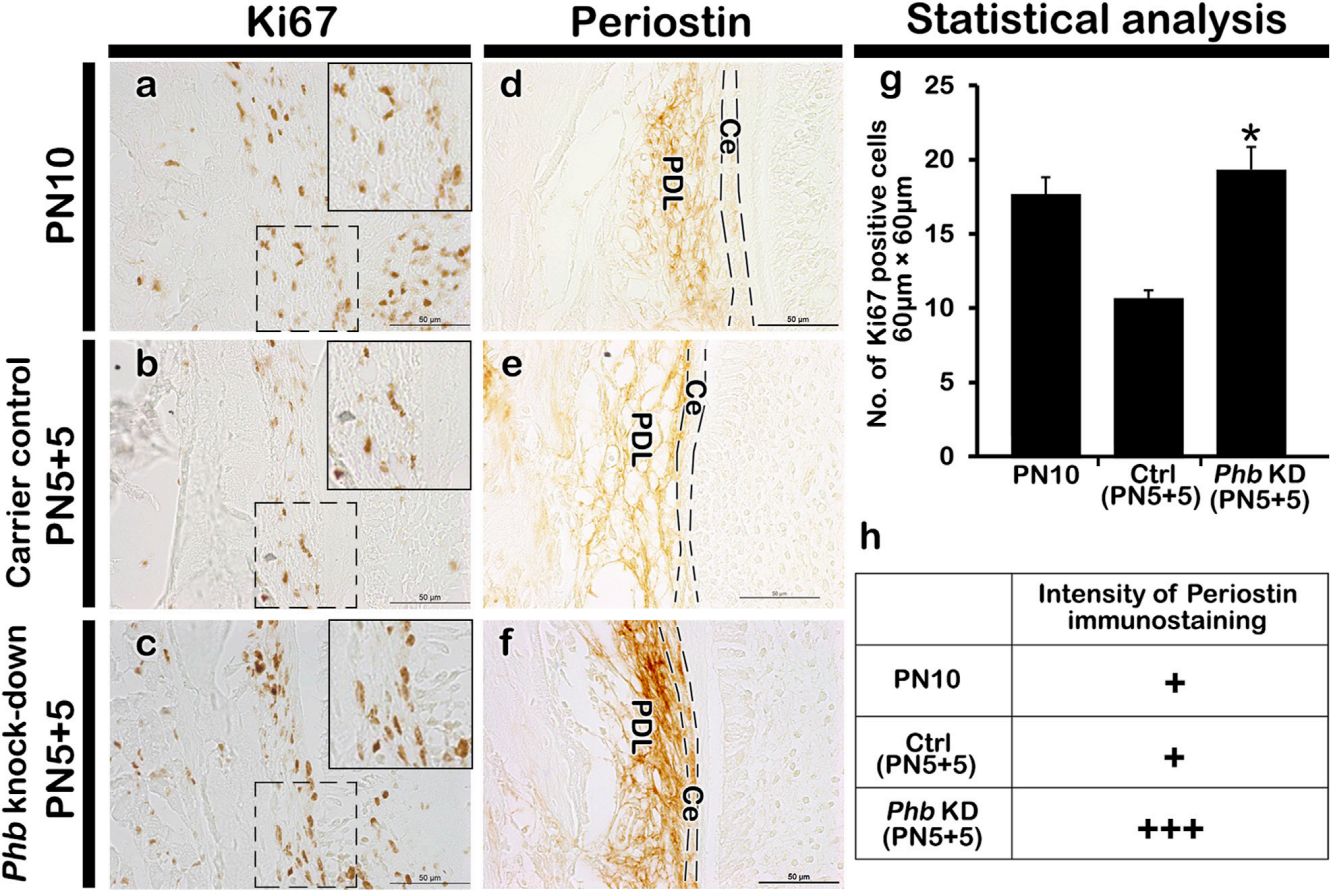
Alteration in cellular physiology and morphogenesis in periodontium. Compared with PN10 and carrier control, *Phb* knock-down shows an increased number of the Ki67 positive cells in PDL forming region **(A–C)**, **(G)**. The localization pattern of PERIOSTIN is observed in the cell layer adjacent to both the alveolar bone and the root sheath **(D–F)**. Compared to controls, a stronger positive reaction against PERIOSTIN is observed in *Phb* knock-down specimens **(D–F)**. The intensity of immunostaining of Periostin is quantified as: none, +: exist, ++: strong, +++: strongest, due to their broad nature of localization patterns outside the nucleus **(H)**. Ce; cementum, PDL; periodontal ligament. Dotted boxes indicate the magnified regions and dotted lines indicate the margin of cementum. *Scale bars*: 50 μm **(A–F)**.

To examine the role of PHB in alveolar bone formation, immunolocalizations of pre-osteoblast transcription factor (RUNX2) and osteoblast (OCN) after *Phb* knock-down were performed (Figures 4aa’-cc’). Our results showed that *Phb* knock-down specimens showed decreased localizations of PHB, RUNX2 and OCN along the PDL and alveolar bone forming regions compared to control (Figures 4aa’a”–cc’c”). Interestingly, the PHB localizations coincide with the localization patterns of RUNX2 and OCN (Figures 4aa’–cc’). On the other hand, the number of osteoclast positive cells were increased in the alveolar bone after *Phb* knock-down (Figure 4dd’d”). Furthermore, we examined changes in cellular proliferation during periodontium development using Ki67 immunohistochemistry (Figure 5). The number of Ki67-positive cells increased after *Phb* knock-down compared to controls (Figures 5A–C,G). Meanwhile, cellular apoptosis remained unchanged in both the control and knock-down specimens (data not shown). Additionally, we investigated PERIOSTIN immunostaining after *Phb* knock-down (Figures 5D–F) because it is a specific protein indicating the maturation level of PDL fibers (Suzuki et al., 2004). PERIOSTIN-positive cells were localized within the fibrous bundles of the PDL (Figures 5D–F). Our results indicated that knocking down *Phb* led to increased PERIOSTIN-positive cells along the fibrous bundles of the PDL compared to controls (Figures 5D–F,H).

### Evaluation of mineralized tissue formation using micro-CT

**FIGURE 6:**
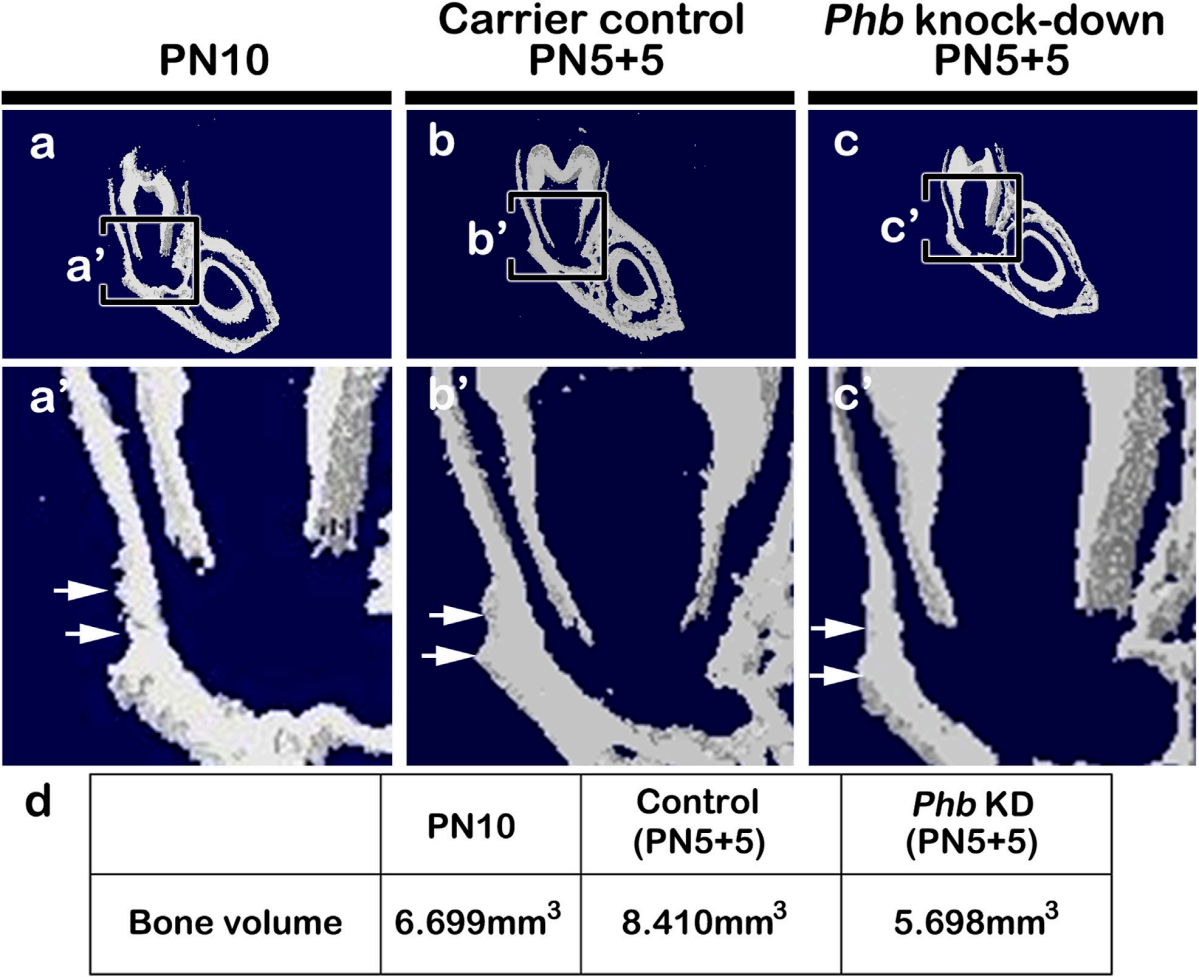
Micro-CT evaluations of hard tissue formation. Micro-CT images of the frontal section of the mandibular first molar at PN10 **(A)**, carrier control **(B)** and *Phb* knock-down specimens **(C)**. Compared to PN10 **(A)** and carrier control **(B)**, the *Phb* knock-down specimen shows the thinner alveolar bone facing the tooth root forming region **(C)**. Arrows indicate the region of interest in the alveolar bone **(B, C)**. The table of analysis using the CT AnalyzerTM software **(D)**.

To understand the developmental role of *Phb* in periodontium formation, we assessed the level of hard tissue formation in the PN10, carrier control (PN5+5) and *Phb* knock-down (PN5+5) specimens using micro-CT (Figure 6). Following *Phb* knock-down, the thickness of the alveolar bone facing the tooth root forming region appeared to decrease compared to the control specimens (Figures 6A–C). The bone volume in the control specimens was higher (PN10: 6.699mm^3^, Carrier control: 8.410 mm^3^) than that in the *Phb* knock-down specimens (5.698 mm^3^), as determined by CT AnalyzerTM software (Skyscan, Kontich, Belgium) (Figure 6D). The carrier control specimen (Figures 6B, Supplementary Figure S2) exhibited a slightly increased mass of alveolar bone forming tissues on the buccal side of the lower molar compared to that of the PN10 (Figure 6A). This increase might be attributed to additional stimulations during micro-injections.

## Discussion

In this study, we utilized previously established micro-injection and *in vitro* tissue culture model systems to evaluate the developmental function of signaling molecules involved in periodontium formation, as previously described (An et al., 2017). Prior research primarily employed experimental studies, including primary cell cultivation and the pathological conditions of animal model systems, focusing solely on and presenting results restricted to the periodontal ligament (Cerri et al., 2000; Sena et al., 2003; Luan et al., 2006; Flores et al., 2008; Fuji et al., 2008; Polimeni et al., 2009; Kaku and Yamauchi, 2014; Neupane et al., 2020). These fragmented findings, limited to the periodontal ligament, are insufficient for comprehensively understanding the developmental and regenerative mechanisms within the entire periodontium. Considering that the periodontium comprises various cell lineages and necessitates coordinated regulations for its development and regeneration, our study employed the aforementioned model systems to elucidate the three-dimensional mechanisms underlying periodontium development and regeneration. This involved examining alteration patterns of morphogenesis in adjacent tissues and expressions of related signaling molecules. Anatomical markers, such as the positions of blood vessels and the oral cavity, were taken into account for the designation of micro-injections, as previously described (An et al., 2017).

Dental follicle cells play a crucial role in giving rise to the periodontal ligament (PDL), alveolar bone, and cementum, establishing spatial patterning during periodontium development (Somerman et al., 1999; Mizuno et al., 2005). The comparative thickness of the tissue forming PDL between the alveolar bone and the regions forming the tooth root undergoes specific alterations, driven by three-dimensional and differential growth of each tissue through specific cellular mechanisms, including lateral inhibition (Kim et al., 2007). The PDL itself exhibits a balanced proliferation and differentiation due to distinct cellular activities. In the mid-region of the PDL, cells initiate the production of extracellular matrix, while concurrently displaying the highest rate of type I collagen expression and differentiation, alongside extensive remodeling and vascularization (Rooker et al., 2010). Drawing from these previous reports, we hypothesized that signaling molecules play a determining role in the precise interactions and pattern formation of the periodontium during its developmental stages. At PN5, the lower molar initiates root development concurrent with the termination of crown development. By PN10, the buccal side of the periodontium of the lower first molar exhibits the evident structural formation of the three components of the periodontium: alveolar bone, periodontal ligament, and cementum (Figures 1, 3). Consequently, the developmental stages at PN5 and PN10 are critical time points for tooth root and periodontium development in mice. Based on observed morphological changes, we selected PN5 for micro-injection and harvested specimens after 5 days at PN10 (Figure 3; An et al., 2017).

*Phb* has been reported to play various roles in transcriptional regulation, cell proliferation, mitochondrial function as well as in uterine development and ovarian cell differentiation (Thompson et al., 2001; Mishra et al., 2005; He et al., 2011; Theiss and Sitaraman, 2011; Supale et al., 2013; Della-Flora Nunes et al., 2021). It also exhibits involvement in the negative regulation of osteoclast differentiation (Lee et al., 2015) and regulates the proliferation and formation of osteoblasts (Zhu et al., 2010). To comprehend the developmental role of PHB in periodontium formation, we initially examined the precise localization pattern of PHB using immunohistochemistry (Figure 1). The specific localization pattern of PHB observed in developing periodontium implies its putative role, particularly during alveolar bone and periodontal ligament (PDL) formation. Upon the knock-down of *Phb*, the decreased alveolar bone volume while the increased width of PDL-forming region was observed (Figures 3, 6). Our results demonstrated that knocking down *Phb* increased the proliferation and differentiation of PDL (Figure 5). Notably, after *Phb* knock-down, PERIOSTIN, typically localized in well-differentiated periodontal ligaments (Horiuchi et al., 1999), exhibited a significant increase (Figure 5). These findings suggest that knocking down *Phb* modulates the morphogenesis of the periodontium by enlarging the PDL space and reducing alveolar bone thickness through the alteration of cellular events in alveolar bone and PDL forming tissues. The reformation of PDL space by *Phb* knock-down might offer a potential treatment strategy for ankylosed teeth. Moreover, the decreased PHB, RUNX2 and OCN localizations, and increased osteoclast cells after *Phb* knock-down suggest the important role of PHB in alveolar bone formation as in previous reports (Figure 4; Zhu et al., 2010; Lee et al., 2015; Tabti et al., 2021).

We also investigated the underlying molecular mechanisms regulated by *Phb* using a drop *in vitro* cultivation method, as previously reported (Jung et al., 2017). At E14.5, dental follicle cells remain undifferentiated and possess the potential to differentiate into the periodontium (Kim et al., 2007). In our examination, we focused on paracrine signaling pathways, including Shh, Bmps, Fgfs, and Wnt, recognized as important signaling molecules during periodontium differentiation (Tummers and Thesleff, 2009). Shh signaling plays a critical role as an epithelial factor for tooth crown and HERS formation, while Fgf signaling is predominantly expressed in dental pulp cells, contributing to cell proliferation and differentiation (Neubuser et al., 1997; Thesleff et al., 2001; Thesleff, 2003; Handrigan and Richman, 2010). Wnt signals stimulate osteogenic transcription factors, initiating the differentiation of periodontal ligament (PDL) fibroblasts into the osteogenic lineage (Rooker et al., 2010; Jung et al., 2017). Furthermore, Wnt signals are responsible for cementoblast maturation (Nemoto et al., 2009) and the maintenance of alveolar bone volume and osteoblasts (Yin et al., 2015). Similarly, BMP signaling regulates the development of calcified tissues by directing the differentiation of mesenchymal precursor cells (Beederman et al., 2013). Based on these results, we suggest that altered signaling molecules provide compelling evidence for the transformed morphogenesis of the periodontium during the developmental phase. The assessment of altered morphogenesis in hard tissue formation was performed using micro-CT image analysis (Figure 6), aligning with previous reports demonstrating the osteogenic potential of PHB (Jung et al., 2017).

In conclusion, employing functional analysis model systems such as the micro-injection model and *in vitro* tissue culture model systems proves to be an appropriate method for examining the coordinated regulation of periodontium development. The periodontium displayed specific alteration patterns in both morphological and molecular aspects following the knock-down of *Phb*. Further studies are needed to investigate the potential applications of PHB in the regeneration of periodontal tissue, aiming to restore functionality in cases of periodontal diseases.

## Data Availability

The datasets presented in this study can be found in online repositories. The names of the repository/repositories and accession number(s) can be found in the article/Supplementary Material.
