# Supplementary material for: Prohibitin modulates periodontium differentiation in mice development
Source: Front Cell Dev Biol. 2024 May 2;12:1369634. doi: 10.3389/fcell.2024.1369634 (PMC11096493; doi:10.3389/fcell.2024.1369634)

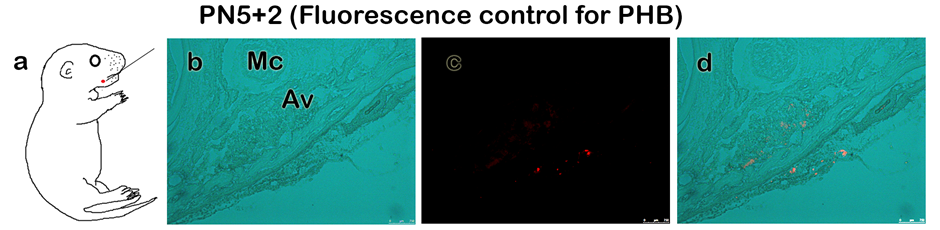


**Figure S1.** Schematic diagram showing micro-injection into the alveolar bone forming region of right mandible at PN5 using Hamilton syringe (a). DiI microinjection at PN5 showing successful microinjection in alveolar bone forming region and its diffusion after 2 days (b-d). Mc, meckel’s cartilage; Av, alveolar bone. * denotes the point of injection.


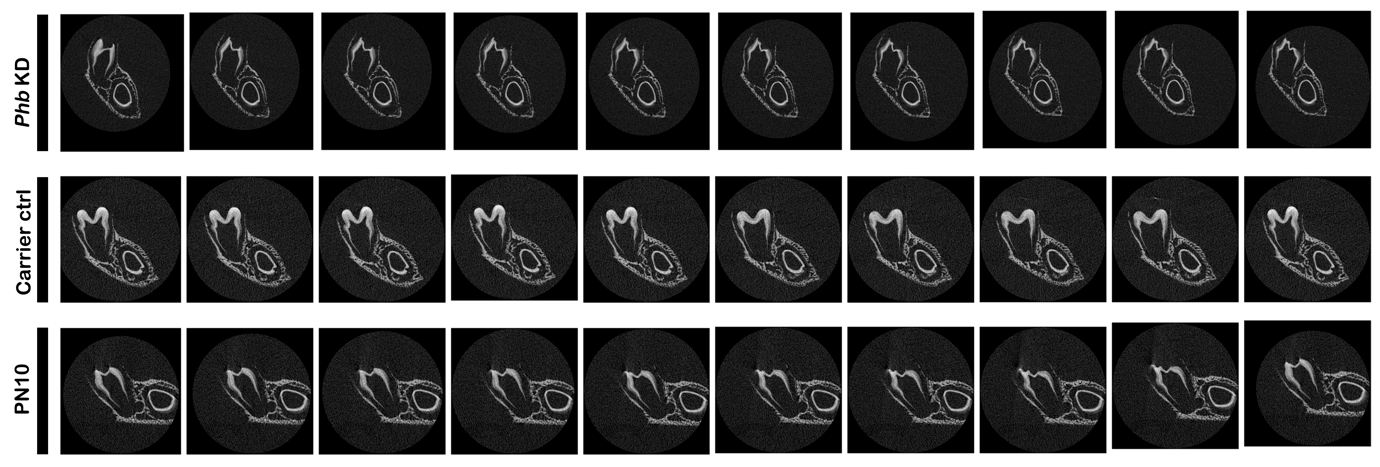


**Figure S2.** Micro-CT images for bone volume analysis

**Table S1.** List of primers used in the study.


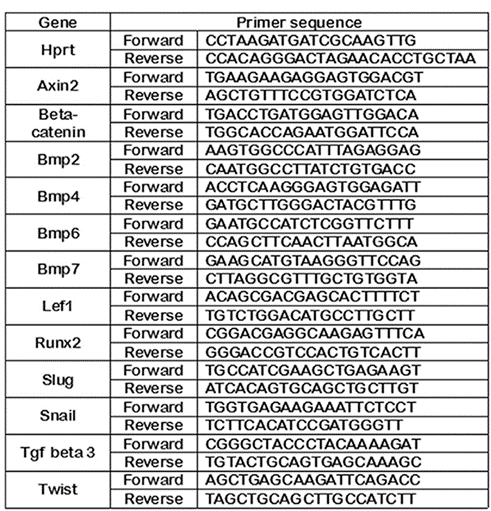

Supplement: Supplementary file 1 [file DataSheet1.docx]
